# An updated checklist of the extant Western Palaearctic Dryininae (Hymenoptera, Dryinidae)

**DOI:** 10.3897/zookeys.874.33226

**Published:** 2019-09-05

**Authors:** Adalgisa Guglielmino, Massimo Olmi, Jing-xian Liu, Mario Contarini

**Affiliations:** 1 Department of Agriculture and Forest Sciences (DAFNE), University of Tuscia, Viterbo, Italy University of Tuscia Viterbo Italy; 2 Tropical Entomology Research Center, Viterbo, Italy Tropical Entomology Research Center Viterbo Italy; 3 Department of Entomology, South China Agricultural University, Guangzhou, China South China Agricultural University Guangzhou China

**Keywords:** Checklist, distribution, Chrysidoidea

## Abstract

A checklist of 20 extant species of Dryininae (Hymenoptera, Dryinidae) from the Western Palaearctic subregion is presented.

## Introduction

Pincer wasps (Hymenoptera, Dryinidae) are parasitoids and often also predators of Auchenorrhyncha (Hemiptera) (Olmi 1984). The family includes 50 genera and 16 subfamilies (Olmi and Xu 2015; Tribull 2015). In the Palaearctic region, the subfamily Dryininae is represented by two extant genera, *Dryinus* Latreille, 1804 and *Pseudodryinus* Olmi, 1991. *Pseudodryinus* is known only from the Eastern Palaearctic subregion and *Dryinus* from both Palaearctic subregions, Eastern and Western (Olmi and Xu 2015).

A review of the Western Palaearctic Dryininae (Hymenoptera, Dryinidae) was published by Olmi (1984), and he listed a total of nine species. However, in the last 25 years many additional papers on the Western Palaearctic fauna have been published, so that the number of species has increased to 20, and the need to develop a new checklist of Western Palaearctic Dryininae became evident. The objective of this checklist is to ease further studies on Palaearctic dryinids.

## Material and methods

The present paper treats all extant Dryininae (fossil species are excluded) present in the Western Palaearctic subregion, i.e., according to Vigna Taglianti et al. (1992, 1999), the part of the Palaearctic region situated in Europe and Asia west to the Ural Mountains and Caspian Sea, from the Azores and Canary Islands to Iran (included). The borders are not always obvious and natural. The eastern boundary runs along the Ural Mountains and the eastern bank of Caspian Sea, reaching Iran. The Russian region situated immediately east of Ural Mountains in parts of Kazakhstan, Turkmenistan, and Iran should be considered transition country to the Eastern Palaearctic subregion, whereas a large part of the Arabian Peninsula should be considered a transition area to the Afrotropical region. All these transition areas are considered in this checklist. The knowledge of the dryinids living in the Western Palaearctic subregion is broadly insufficient, so that this checklist will need to be updated in the future following further research.

Distributional data of Dryininae in the Western Palaearctic region were compiled analysing all the available publications, in addition to many unpublished records obtained by identifying material belonging to various institutions.

All the localities cited in this checklist, except that from Belarus cited by Shlyakhtyenok (2013) (see *Dryinus collaris* (Linnaeus)), were checked by the authors by examining personally all the specimens. The examined specimens are deposited in the following collections:

**AEC** Christoph Saure’s collection, Berlin, Germany.

**AMNH**American Museum of Natural History, New York, USA.

**ASM** Alexander Shlyakhtenok’s collection, Minsk, Belarus.

**BNC** Benoît Nusillard’s collection, Montboucher sur Jabron, France.

**BWC** Bogdan Wiśniowski’s collection, Ojców National Park, Ojców, Poland.

**CAS**California Academy of Sciences, San Francisco, California, USA.

**CIRAD**Centre International de Recherche Agricole pour le Développement, Montpellier, France.

**CNC**Canadian National Collection of Insects (CNCI), Ottawa, Canada.

**DEI**Senckenberg Deutsches Entomologisches Institut, Müncheberg, Germany.

**DEUW** Department of Entomology, University of Wageningen, the Netherlands.

**DISAFA** Dipartimento di Scienze agrarie, forestali e alimentari, University of Torino, Grugliasco, Torino, Italy.

**DPPZ** Department of Plant Protection, College of Agriculture, University of Zabol, Iran.

**ENSAM**École National Supérieure Agronomique, Montpellier, France.

**FBW** Forstliche Versuchs- und Forschungsanstalt Baden-Württemberg, Freiburg, Germany.

**FSAE** Faculté des Sciences Agronomiques de l’État, Gembloux, Belgium.

**GLPC** Gianluca Parise’s collection, Carignano, Torino, Italy.

**GNC** Göran Nilsson’s collection, c/o Department of Zoophysiology, Uppsala University, Uppsala, Sweden.

**GPC** Guido Pagliano’s collection, Torino, Italy.

**HMO** Hope Museum, Oxford, England, United Kingdom.

**HTS** Hubert Tussac’s collection, Cahors, Lot, France (now c/o Museum d’Histoire naturelle, Genève, Switzerland).

**IGC** Ilia Gjonov’s collection, Sofia, Bulgaria.

**IRSN**Institut Royal de Sciences Naturelles de Belgique, Bruxelles, Belgium.

**JBZC** Javier Blasco-Zumeta’s collection, Pina de Ebro, Zaragoza, Spain.

**JTBC** John T. Burn’s collection, Sacriston, England, United Kingdom.

**LOHC** Lars Ove Hansen’s collection, Drammen, Norway.

**MBC** Manuel Baena’s collection, Cordoba, Spain.

**MCNTN** Museo de Ciencias Naturales, Santa Cruz, Tenerife, Canary Islands, Spain.

**MCSNG** Museo Civico di Storia Naturale “Giacomo Doria” di Genova, Italy.

**MCSNV**Museo Civico di Storia Naturale, Verona, Italy.

**MHNG**Muséum d’Histoire Naturelle, Genève, Switzerland.

**MLUHW**Martin-Luther-Universität, Halle-Wittenberg, Germany.

**MNCNM**Museo Nacional de Ciencias Naturales, Madrid, Spain.

**MNHN**Muséum National d’Histoire Naturelle, Paris, France.

**MMB**Moravian Museum, Brno, Czech Republic.

**MOLC** Massimo Olmi’s collection, Viterbo, Italy.

**MSC** Massimiliano Spinola’s collection, c/o Museo Regionale di Scienze Naturali, Torino, Italy.

**MSCS** Martin Schwarz’s collections, c/o Institut für Zoologie, Salzburg, Austria.

**MSNTC**Museo di Storia naturale e del Territorio, Università di Pisa, Calci, Italy.

**MZUN** Museo di Zoologia dell’Università, Napoli, Italy.

**NHMUK**Natural History Museum, London, United Kingdom.

**NMNH** National Museum of Natural History, Budapest, Hungary.

**NMPC**National Museum (Natural History), Praha, Czech Republic.

**NMW** Naturhistorischen Museum, Wien, Austria.

**OLL**Oberösterreichisches Landesmuseum, Linz, Austria.

**PNL** Pierre-Nicolas Libert’s collection, Somme-Leuze, Belgium.

**RNHL** Rijksmuseum van Natuurlijke Historie, Leiden, The Netherlands.

**SVC** Simo Väänänen’s collection, Vantaa, Finland.

**SZC** Pier Luigi Scaramozzino’s collection, Pisa, Italy.

**USNM**National Museum of Natural History, Washington, DC, USA.

**VVC** Veli Vikberg’s collection, Turenki, Finland.

**WHC** Paul Whitehead’s collection, Moor Leys, England, United Kingdom.

**YUIC**Yeungnam University Insect Collection, Department of Biology, Yeungnam University, Kyongsan, South Korea.

**ZIL** Zoological Institute, Lund, Sweden.

**ZMK**Zoologisk Museum, Copenhagen, Denmark.

**ZMM** Zoological Museum of Moscow University, Moscow, Russia.

**ZMUH** Zoological Museum of the University, Helsinki, Finland.

## Checklist of the extant Western Palaearctic Dryininae Haliday, 1833

### Genus *Dryinus* Latreille, 1804

#### 1. *Dryinus albrechti* (Olmi)

*Richardsidryinus albrechti*Olmi 1984: 909.

*Dryinus albrechti* (Olmi): Olmi 1999: 192.

**SPAIN**: **Canary Islands**: Fuerteventura, Las Peñitas (MNCNM) (Olmi 1984); Lanzarote, El Risco de Famara (AMNH) (Olmi 1984); Tenerife, Orotava (ZMUH) (Olmi 1984).

**Distribution**: Spain.

#### 2. *Dryinus balearicus* Olmi

*Dryinus balearicus*Olmi 1987: 418; Olmi 1999: 209.

**SPAIN: Balearic Islands**: Ibiza, 5 km N San José (NHMUK, AMNH) (Olmi 1987). **Continental Spain**: Huesca Prov., near Torla, Fanlo (NHMUK). **TUNISIA**: Barrage Mellègue (MOLC).

**Distribution**: Spain, Tunisia.

#### 3. *Dryinus berlandi* (Bernard)

*Chelothelius berlandi*Bernard 1935: 41; Olmi 1984: 609.

*Dryinus berlandi* (Bernard): Olmi 1999: 202.

**FRANCE**: Var, Fréjus, Saint-Raphaël beach (MNHN) (Bernard 1935). **MOROCCO**: along Road P 39, 69 km Melilla, Dar Driouch (MBC) (Olmi 1999). **TUNISIA**: 10 km N of Jendouba (OLL).

**Distribution**: France, Morocco, Tunisia.

#### 4. *Dryinus canariensis* (Ceballos)

*Paradryinus canariensis*Ceballos 1927: 101.

*Dryinus canariensis* (Ceballos): Olmi 1984: 734; 1999: 184.

**SPAIN: Canary Islands**: Gomera, San Sebastian, Barranco de Marchar (MNCNM) (Olmi 1984); Gomera, Chejelipes (NHMUK); Hierro, Frontera (MCNTN); La Palma, Montaña Brena (OLL); Tenerife, Barranco Santos (AMNH, MCNTN, MNCNM) (Olmi 1984); Tenerife, Desembocadura del Barranco de Tejina (AMNH); Tenerife, La Cuesta (MNCNM) (Ceballos 1927); Tenerife, Tahodio (MNCNM) (Olmi1984); Tenerife, Médano, Los Calderones (MCNTN, MNCNM) (Olmi 1984); Tenerife, Médano (MNCNM); Tenerife, Arico, Montaña Atalaya (MNCNM); Tenerife, Bajamar (MNCNM); Tenerife, Las Mercedes (MNCNM); Tenerife, Carretera de San Andres, Jagua (AMNH); Tenerife, Raguo Negro (OLL). **EGYPT**: Sinai, Saint Catherine area (MOLC). **GREECE**: Rhodes Island, ridge N of Psinthos (NHMUK).

**Distribution**: Egypt, Greece, Spain.

#### 5. *Dryinus collaris* (Linnaeus)

*Sphex collaris*Linnaeus 1767: 946.

*Dryinus formicarius*Latreille 1805: 228 (synonymized by Fitton et al. 1978).

*Campylonyx ampuliciformis*Westwood 1835: 52 (synonymized by Olmi 1984).

*Lestodryinus formicarius* (Latreille): Kieffer 1914a: 20.

*Lestodryinus corsicae*Kieffer 1914a: 21 (synonymized by Olmi 1984).

Dryinus (Lestodryinus) formicarius Latreille: Haupt 1932: 15.

*Dryinus collaris* (Linnaeus): Olmi 1999: 185; Olmi and Xu 2015: 133.

**AUSTRIA**: Niederösterreich, Piesting (NMW); Oberösterreich, Hinteraigen, E Aibach/Donau, 48°24'N, 13°57'E (OLL); Salzburg, Werfen (RNHL) (Olmi 1984); Steiermark, O-Steiermark, E Weiz, Hoferberg (OLL); Wien, Dornbach (MNHN) (Olmi 1984). **BELARUS**: Polyese Radiacyonno-ekologichesky zapovednik, Dronki (ASM) (Shlyakhtyenok 2013). **BELGIUM**: Brabant, Forêt de Soignes (IRSN); Liège, Flémalle-Haute aux Roches (IRSN); Namur, Ave-et-Auffe, Thérimont (IRSN); Namur, Somal (PNL). **CROATIA**: Istra, Opatija (NMNH) (Olmi 1984); Krapina (NMNH) (Olmi 1984). **FRANCE**: Alpes de Haute-Provence, Digne (MNHN) (Olmi 1984); Corse (ENSAM) (Olmi 1999); Gard, Bez-et-Esparon (CIRAD) (Tussac and Olmi 1998); Haute-Garonne, Clermont-le-Fort (HTS) (Tussac and Olmi 1998); Haute-Garonne, Toulouse, L’Isle-Jourdain (FSAE); Haute-Loire, Le Puy (MNHN) (Olmi 1984); Haute-Savoie, Bossy-Frangy (MHNG); Hérault, Saint-Gély-du-Fesc (MNHN) (Olmi 1984); Hérault, Mons-la-Triviale, Gorges d’Héric (CIRAD) (Tussac and Olmi 1998); Hérault, Saint-Guilhem-le-Désert (MNHN) (Olmi 1984); Landes, Mont-de-Marsan (MNHN) (Olmi 1984); Pyrénées-Orientales, Forêt de Boucheville (DEUW); Rhône, Lyon (MNHN) (Westwood 1835); Saône-et-Loire, Les Gerraux (MNHN) (Olmi 1984); Var, Hyères (MNHN) (Olmi 1984); Var, Toulon (MNHN) (Olmi 1984); Var, Sainte-Baume (MNHN); Vaucluse, near Bédoin (NHMUK). **GERMANY**: Baden-Württemberg, Baden, Freiburg i. B., Bechtaler Wald, 48°12'N, 07°42'E (FBW); Baden-Württemberg, Freiburg im Breisgau, Mooswald-Nord (AEC); Nordrhein – Westfalen, Aix-la-Chapelle (= Aachen) (Kieffer and Marshall 1905). **HUNGARY**: Borsod-Abaúj-Zemplén county, Cserépfalu, Hór-völgy (NMNH, AMNH) (Szöllösi-Tóth and György 2009). **ITALY**: Campania, Napoli Prov., Napoli (Kieffer and Marshall 1905); Emilia Romagna, Bologna Prov., Gaibola (AMNH) (Olmi 1984); Liguria, Genova Prov., S. Olcese (NHMUK) (Olmi 1984); Piemonte, Cuneo Prov., Valmala, along Comba di Valmala, Ponte Parasacco (IRSN); Piemonte, Torino Prov., Rosta (AMNH) (Olmi 1984); Piemonte, Torino Prov., Strambino (DISAFA) (Olmi 1999); Puglia, Taranto Prov., Mottola, S. Basilio (Móczár 1965); Sicilia, Catania Prov., Bronte, Maletto, Mt. Etna, Contrada Paviglione (AMNH) (Olmi 1999); Toscana, Lucca Prov., Lucca (Kieffer and Marshall 1905); Toscana, Lucca Prov., Lido di Camaiore (MOLC) (Olmi 2005b); Trentino Alto Adige, Bolzano Prov., Bolzano (Schmiedeknecht 1907). **MONTENEGRO**: Herceg Novi (= Castelnuovo di Cattaro) (DEI); Zelenika (NMNH) (Olmi 1984). **POLAND**: East bank of Oder River, 10 km N of Cedynia, Bielinek (= Bellinchen) (Haupt 1932, as Dryinus (Lestodryinus) formicarius). **SLOVAKIA**: SW Slovakia, Little Carpathians (Malé Karpaty), near confluence Danube and Morava Rivers, Devínska Kobyla Hill (Lukás 1998). **SPAIN**: **Balearic Islands**: Mallorca, Porto Cristo (RNHL). **Continental Spain**: Alicante Prov., Sierra de Altana (RNHL). **SWITZERLAND**: Genève, Peney (MHNG) (Olmi 1984); Genève (MSC) (Olmi 1984); Genève, Bois de Collex (MHNG); Genève, Place des Nations (NMNH); Valais, Châteauneuf (MHNG); Ticino, Gandria (NMNH, AMNH) (Olmi 1999). **THE NETHERLANDS**: Lexmond (RNHL); Neercanne, Cannerbos (RNHL) (De Rond 2004). **TURKMENISTAN** (Ponomarenko 1978). **UNITED KINGDOM**: **England**: Berkshire, High Standinghill Wood, Windsor Forest (NHMUK); Middlesex, Ruislip, Victoria Road (NHMUK); Surrey, Banstead Downs (NHMUK); Surrey, Shere (Capron 1885); Surrey, Reigate, 30 Park Lane East (only photographed, not collected); West Kent, Cobham (Richards 1939); West Kent, Eltham (JTBC); Worcestershire, Malvern Hills (WHC) (Whitehead 2010).

**Distribution**: Austria, Belarus, Belgium, Croatia, France, Germany, Hungary, Italy, Montenegro, Poland, Slovakia, Spain, Switzerland, the Netherlands, United Kingdom, in addition to Turkmenistan (transition country to Eastern Palaearctic subregion).

#### 6. *Dryinus corsicus* Marshall

*Dryinus corsicus*Marshall 1874: 207; Olmi and Xu 2015: 133.

*Mesodryinus corsicus* (Marshall): Kieffer 1907: 10.

*Mesodryinus escorialensis*Ceballos 1927: 102 (synonymized by Olmi 1984).

*Richardsidryinus corsicus* (Marshall): Móczár 1965: 377.

**CYPRUS**: Limassol (NHMUK) (Olmi 1984). **FRANCE**: Alpes-Maritimes, Breil-sur-Roya, Col des Termes (CIRAD); Aude, Brouilla (BNC); Bouches-du-Rhône, Aix-en-Provence (NHMUK) (Olmi 1984); Corse, Ajaccio, Campoloro (NMNH) (Olmi 1984); Drôme, Montségur-sur-Lauzon (HTS); Drôme, Mévouillon (BNC); Drôme, S.te Jalle (RNHL); Drôme, Séderon, Col de l’Homme mort (AMNH); Drôme, Col de Macuègne (NHMUK); Gironde, Barsac (HTS) (Tussac and Olmi 1998); Hérault, Cazevieille (MNHN) (Tussac and Olmi 1998); Hérault, Montpellier (HTS) (Tussac and Olmi 1998); Hérault, Grabels (HTS); Hérault, La Figarède (MNHN) (Olmi 1984); Hérault, St. Gély-du-Fesc (MNHN) (Olmi 1984); Hérault, Balliarguet CSIRO, 43°41.12'N, 03°62.24'E (CNC); Haute-Garonne, Castelmaurou (HTS) (Tussac and Olmi 1998); Lot, Cahors (HTS) (Tussac and Olmi 1998); Lot, Le Montat (HTS); Var, near St. Zacharie (NHMUK); Vaucluse, Sérignan (MNHN) (Olmi 1984); Vaucluse, Lagarde d’Apt, Mt St Pierre (BNC). **GREECE**: Olympia, Ilia (NHMUK) (Olmi 1984); Peloponìsos, Monemvasia (ZMK). **HUNGARY**: Somogy county, Kaposvár (NMNH) (Olmi 1984). **ITALY**: Calabria, Crotone Prov., Sila, along road from Pagliarelle to Mt. Gariglione, about 9.7 km from Pagliarelle, 39°07.382'N, 16°41.553'E (MOLC); Friuli Venezia Giulia, Trieste Prov., Villa Opicina (DEI) (Olmi 1984); Emilia Romagna, Forlì Prov. Campigna Forest (MCSNV) (Olmi 1999); Toscana, Pisa Prov., Lajatico, 43°27.86'N, 10°40.73'E (MOLC) (Olmi 2005b). **KAZAKHSTAN**: Tchimkent obl., Karatau Ridge near Suzak (ZMM) (Ponomarenko and Olmi 2006). **SPAIN**: Barcelona, Palamos (SZC) (Olmi 1984); Murcia, near Manzarron (NHMUK) (Olmi 1984); Murcia, Sierra de Espuña, near Totana (NHMUK) (Olmi 1984); Madrid, El Escorial (MNCNM) (Ceballos 1927); Granada, Cubillas (AMNH, NHMUK) (Olmi 1984); Granada, Nerja (NHMUK); Castellon, Benicasim (NHMUK); 10 km from Abejar, Soria (RNHL); Alicante, Jávea (HTS); Zaragoza, Pina de Ebro, Monegros (HTS, JBZC) (Olmi et al. 1998).

**Distribution**: Cyprus, France, Greece, Hungary, Italy, Spain, in addition to Kazakhstan (transition country to Eastern Palaearctic subregion).

#### 7. *Dryinus dayi* (Olmi)

*Mesodryinus dayi*Olmi 1984: 1003.

*Dryinus dayi* (Olmi): Olmi 1999: 204.

**GREECE**: Thessalia, Kalambaka (NHMUK) (Olmi 1984).

**Distribution**: Greece.

#### 8. *Dryinus delvarei* Olmi

*Dryinus delvarei*Olmi 1998: 72.

**ALBANIA**: Mirditë District, Salitë (MOLC). **ITALY**: Toscana, Arezzo Province, Upacchi, 43°30'N, 11°59'E (MSCS); Toscana, Grosseto Prov., Maremma Natural Park, 42°38.44'N, 11°04.42'E (MSNTC) (Olmi 2005b). **TURKEY**: 18 km NW Korkuteli (AMNH) (Olmi 1998).

**Distribution**. Albania, Italy, Turkey.

#### 9. *Dryinus gharaeii* Olmi

*Dryinus gharaeii*Olmi 2005a: 207; Olmi and Xu 2015: 144.

**IRAN**: Ilam Province, Chogasabz Region, Ilam (MOLC) (Olmi 2005a).

**Distribution**. Iran.

#### 10. *Dryinus gryps* (Reinhard)

*Chelothelius gryps*Reinhard 1863: 410.

*Dryinus gryps* (Reinhard): Dalla Torre 1898: 544; Olmi 1995: 5.

**FRANCE**: Bouches-du-Rhône, Fonscolombe (NHMUK); Drôme, Montségur-sur-Lauzon (BNC); Gard, Ussel-Goudargues (AMNH) (Olmi 1984); Hérault, Montagnac, Mas de Linares (HTS) (Tussac and Olmi 1998); Lot, Cahors (HTS); Southern France (MNHN) (Olmi 1984). **ITALY**: Sicilia, Siracusa Province, Lentini (AMNH) (Olmi 1999); Toscana, Livorno Province, near Piombino, Salivoli, 42°56.79'N, 10°30.20'E (MOLC) (Olmi 2005b); Toscana, Pisa Province, Monteverdi Marittimo, 43°09.59'N, 10°43.24'E (MOLC) (Olmi 2005b); Trentino-Alto Adige, Bolzano (Reinhard 1863). **SPAIN**: Zaragoza, Pina de Ebro, Los Monegros (HTS) (Olmi et al. 1998); Madrid, El Pardo (MNCNM); Cataluña, Tarragona, El Perello (HTS). **TURKEY**: Konya, Meram (OLL).

**Distribution**. France, Italy, Spain, Turkey.

#### 11. *Dryinus ibericus* (Olmi)

*Alphadryinus ibericus*Olmi 1990: 137.

*Dryinus ibericus* (Olmi): Olmi 1999: 208.

**SPAIN**: Murcia, Albacete Prov., near Molinicos, El Pardal (MNHN) (Olmi 1990); Granada, Sierra de Cazorla, Vacillo (HTS) (Olmi 1999).

**Distribution**. Spain.

#### 12. *Dryinus maroccanus* (Olmi)

*Richardsidryinus maroccanus*Olmi 1984: 910.

*Dryinus maroccanus* (Olmi): Tussac and Olmi 1998: 488; Olmi 1999: 193.

**ALGERIA**: Oran (MHNG, MOLC) (Olmi 1984). **FRANCE**: Alpes-Maritimes, Valbonne (BNC); Hérault, Cazevieille (CIRAD) (Olmi 1999). **MOROCCO**: Tangeri (MHNG) (Olmi 1984). **SPAIN**: Madrid, El Pardo, El Goloso (AMNH) (Olmi 1999).

**Distribution**. Algeria, France, Morocco, Spain.

#### 13. *Dryinus niger* Kieffer

*Dryinus niger*Kieffer 1904: 352; Olmi 1999: 206.

*Mesodryinus niger* (Kieffer): Kieffer and Marshall 1906: 497; Olmi 1984: 1005.

*Mesodryinus brittanicus*Richards 1939: 228 (synonymized by Richards 1953).

**ALBANIA**: Arras, 10 km NW Peshkopi (OLL) (Olmi 1994). **CYPRUS**: Cherkes (NHMUK) (Olmi 1984). **CZECH REPUBLIC**: Central Bohemia, Celakovice, Lipovka (NMPC) (Macek 2007); Oriental Bohemia, Zelezné hory PLA, Zlatá louka National Reserve (NMPC) (Macek 2007). **DENMARK**: South Jutland, Sotrup (ZMK) (Olmi 1994). **FINLAND**: Satakunta, Eurajoki (Väänänen and Vikberg 2007) (SVC, VVC). **FRANCE**: Haute-Garonne, Castelmaurou (AMNH, MNHN) (Tussac and Olmi 1998); Lot, Lavercantière (HTS) (Tussac and Olmi 1998); Lot, Cahors (HTS); Vaucluse, Mont Ventoux, Malaucène (MNHN). **GERMANY**: Rheinland-Pfalz, Gönnersdorf (Cölln and Sorg 2001). **GREECE**: Peloponnesus, Monemvasia (ZMK). **ITALY**: Campania, Salerno Prov., Vallo della Lucania (MCSNG) (Kieffer 1904); Liguria, Genova (MCSNG) (Olmi 1984); Piemonte, Cuneo Prov., Valdieri (AMNH) (Olmi 1999); Piemonte, Vercelli Prov., Piode, Alpe Meggiana (MOLC). **NORWAY**: Inner Telemark, Notodden, Lisleherad (LOHC) (Hansen and Olmi 1996; Olmi 1994). **SLOVAKIA**: Southern Slovakia, Senec, Cierna voda river (NMPC) (Macek 2007). **SWEDEN**: Småland (ZIL) (Olmi 1994); Värmland, Ekshärad (CNC) (Olmi 1984); Västmanland, Kärrbo, Solbacken (GNC) (Olmi 1994). **THE NETHERLANDS**: Zuid Holland, Lexmond (De Rond 2004); Gelderland, Kesteren, Lienden (De Rond 2004). **UNITED KINGDOM**: **England**: Dorset, Glanvilles Wootton, f# holotype of *M. brittanicus* (HMO) (Richards 1939); Northants, Ayno, 21–25.VI.1945, R.B. Benson leg., 1f# (NHMUK) (Olmi 1984); Oxfordshire, Otmoor, 19.VII.1961, M.W.R. de V. Graham leg., 1f# (NHMUK).

**Distribution**. Albania, Cyprus, Czech Republic, Denmark, Finland, France, Germany, Greece, Italy, Norway, Slovakia, Sweden, The Netherlands, United Kingdom.

#### 14. *Dryinus sanderi* Olmi

*Dryinus sanderi*Olmi 1984: 731; Olmi 1999: 211.

*Alphadryinus sanderi* (Olmi): Olmi 1991: 284.

**BULGARIA**: Melnik (AMNH) (Olmi 1984); Sandanski (= Liljanovo) (MMB); Upper Thracian Plain, Besapari hills, Novo selo vill., 42.0974N, 24.4690E (IGC) (Lapeva-Gjonova et al. 2018). **CYPRUS**: 10 km W Cape Gréko, Ayia Napa (ZMK). **FRANCE**: Alpes-Maritimes, Moulinet, Sentier Col de Turini-Faysset, 43°58.51'N, 07°24.40'E (CIRAD); Drôme, Séderon, Col de l’Homme mort (AMNH) (Olmi 1999); Hérault, Grabels (HTS) (Tussac and Olmi 1998). **ITALY**: Piemonte, Torino Prov., Susa, Giaglione (AMNH) (Olmi 1999). **RUSSIA**: **European Russia**: Orenburg District, Orsk (Ponomarenko 1992: as *Richardsidryinus albrechti* Olmi).

**Distribution**. Bulgaria, Cyprus, France, Italy, Russia.

#### 15. *Dryinus tamaricicola* Rakhshani & Olmi

*Dryinus tamaricicola* Rakhshani and Olmi in Derafshan et al. 2016: 412.

**IRAN**: Sistan and Baluchestan Prov., Zabol County, Zabol (MOLC) (Derafhsan et al. 2016).

**Distribution**. Iran.

#### 16. *Dryinus tarraconensis* Marshall

*Dryinus tarraconensis*Marshall 1868: 204; Olmi 1984: 742.

*Dryinus szepligetii* Kieffer in Kieffer and Marshall 1905: 77 (synonymized by Olmi 1984).

*Plastodryinus szepligetii* (Kieffer): Kieffer and Marshall 1906: 496.

*Lestodryinus tarraconensis* (Marshall): Kieffer 1914a: 21.

*Lestodryinus gregori*Hoffer 1936: 164 (synonymized by Móczár 1965).

*Lestodryinus bidens*Haupt 1937 (synonymized by Olmi 1984).

*Dryinus szepligetii* Nec Kieffer: Ponomarenko 1981: 879.

**BULGARIA**: Damianitsa, 8 km S of Sandanski (CAS); Sandanski (= Liljanovo) (MMB); Slnoev Brjag (OLL); Nessebar (AMNH); Mt. Strandzha, Izgrev village, 42°08.41'N, 27°48.37'E (IGC). **CROATIA**: Dalmatia, Novi (NMNH) (Olmi 1984). **CZECH REPUBLIC**: South Moravia, Pouzdranyi (Hoffer 1936; Móczár 1965); Moravia, Kobylí (OLL); Moravia, S of Brno, Bratcice, 49°03'N 16°31'E (OLL); Moravia, Havraniky, Znojmo, 48°49'N, 15°59'E (OLL). **FRANCE**: Alpes de Haute-Provence, Simiane-la-Rotonde (HTS); Aude, Salles d’Aude (NMNH); Bouches-du-Rhône, Fonscolombe (NHMUK); Gironde, Barsac (HTS) (Tussac and Olmi 1998); Loiret, Orléans (MNHN) (Olmi 1984); Lot, Cahors (HTS) (Tussac and Olmi 1998); Haute-Garonne, Castelmaurou (HTS) (Tussac and Olmi 1998); Hérault, Grabels (HTS); Hérault, Montpellier (HTS) (Tussac and Olmi 1998); Hérault, Selagou Lake (NHMUK); Vaucluse, near St. Didier, Grange Neuve (NHMUK); Vaucluse, Les Constants, near Bédoin (NHMUK). **GERMANY**: Baden-Württemberg, Mühlacker-Mühlhausen (De Rond, pers. comm.; see Olmi and De Rond 2001). **GREECE**: Corfu Island, Kato Karakiana (JTBC) (Burn 2011); Rhodes Island, Kremasti Hills (NHMUK) (Olmi 1984); Rhodes Island, Ixia (NHMUK); Chalkidiki Peninsula, Amoliani Island (MCSNG). **HUNGARY**: Veszprém county, Balatonkenese (NMNH) (Olmi 1984); Crkvenica (AMNH) (Olmi 1984); Nógrád county, Ipolytarnóc (NMNH) (Szöllösi-Tóth and György 2009); Kiskunság National Park, Bugac puszta (AMNH). **IRAN**: Kerman Prov., Bam County, Sangemes, 28°56'33.44"N, 58°07'52.36"E (DPPZ) (Derafshan et al. 2016); Kermanshah Prov., Kermanshah, Moghoye, 28°57'24.18"N, 58°06'34.90"E (DPPZ) (Derafshan et al. 2016). **IRAQ**: Baghdad (OLL, USNM) (Olmi 1984). **ITALY**: Abruzzi, Pescara Prov., Mt. Maiella (MZUN) (Olmi 1984); Calabria, Crotone Prov., N of Petilia, near road to Pagliarelle, 39°07.333'N, 16°47.036'E (MOLC); Emilia Romagna, Parma Prov., Parma (MZUN) (Olmi 1984); Lazio, Viterbo Prov., Capodimonte (AMNH) (Olmi 1999); Lazio, Viterbo Prov., Sutri (MOLC); Liguria, La Spezia Prov., Vernazza, 44°08.36'N, 09°41.73'E (MOLC); Liguria, Savona Prov., Pietra Ligure (GPC) (Olmi 1984); Piemonte, Alessandria Prov., Gavi (MCSNG) (Olmi 1984); Piemonte, Alessandria Prov., Montaldo di Cerrina (GLPC); Piemonte, Cuneo Prov., Valdieri, *Juniperus phoenicea* Reserve (AMNH) (Olmi 1999); Cuneo Prov., S. Benedetto Belbo (AMNH) (Olmi 1984); Torino Prov., Carignano (Guglielmino et al. 2015); Puglia, Lecce Prov., S. Maria di Leuca Cape (MNHN) (Olmi 1999); Puglia, Taranto Prov., Castellaneta, Bosco dei Terzi, 40°41'26,6"N, 16°57'22,9"E (MOLC); Sardegna, Sassari Prov., Luras (MOLC) (Olmi 2005b); Sicilia, Caltanissetta Prov., S. Cataldo (AMNH) (Olmi 1984); Sicilia, Catania Prov., M. Etna, Bronte, Maletto, Contrada Paviglione (AMNH) (Olmi 1999); Sicilia, Messina Prov., Messina (ZMK) (Olmi 1984); Toscana, Grosseto Prov., Natural Park of Maremma, 42°38.44'N, 11°04.42'E (MOLC) (Olmi 2005b); Toscana, Livorno Prov., Venturina (MOLC) (Olmi 2005b); Toscana, Livorno Prov., Capraia Island (MOLC) (Olmi 2005b); Toscana, Pisa Prov., San Rossore, 43°41'N 10°39'E (MOLC) (Olmi 2005b); Toscana, Pisa Prov., Cipollini (MOLC) (Olmi 2005b); Toscana, Pisa Prov., Monteverdi Marittimo, 43°09.59'N, 10°43.24'E (MOLC) (Olmi 2005b); Toscana, Pisa Prov., Lajatico, 43°27.86'N, 10°40.73'E (MOLC) (Olmi 2005b); Toscana, Pistoia Prov., Montecatini Terme (AMNH) (Olmi 1984); Umbria, Perugia Prov., Perugia (MCSNG) (Olmi 1984); Valle d’Aosta, Aosta Prov., Sarre (NHMUK) (Olmi 1984). **MONTENEGRO**: Sutomore (NMNH) (Olmi 1984); near Kotor, Krasici (NHMUK). **MOROCCO**: High Atlas, 25 km N of Taroudant, Sebt Tafraoute (NHMUK) (**new record**). **POLAND**: East bank of Oder River, 10 km N of Cedynia, Bielinek (= Bellinchen) (MLUHW, MNHN) (Haupt 1937); Wyzyna, Malopolska, Rzezusnia k/Golczy (BWC). **ROMANIA**: Transilvania, Nagyenyed (NMNH). **RUSSIA**: **European Russia**: Volgograd District (Ponomarenko 1978); **Far East**: Primorskij Kraj, Evseyevka, 15 km SE of Spassk (Ponomarenko 1992). **SLOVAKIA**: South Slovakia, Stiavnické, Hronsky Benadik (MMB). **SOUTH KOREA**: GB, Gyeongsan-si, Dae-dong, Yeungnam-Univ., 35°58'N 128°47'E (YUIC). **SPAIN**: Huesca, Torla (NMNH) (Marshall 1868); Madrid, El Escorial (NHMUK) (Olmi 1984); Madrid, El Pardo, El Goloso (MNCNM); Zaragosa, Pina de Ebro, Monegros (HTS) (Olmi et al. 1998); Castellon, Benicasim (NHMUK) (Olmi 1984); Tarragona, Salou (AMNH, NHMUK) (Olmi 1984); Granada, Sierra Nevada (AMNH, CNC); Alicante, Calpe (HTS); Almeria, Carboneras (RNHL); Soria, Ucero (RNHL) . **TAJIKISTAN**: Kulyab obl., 20 km ENE Pyandzh (ZMM) (Ponomarenko and Olmi 2006). **TURKEY**: Urfa, Halfeti (RNHL); Mugla, Köycegiz (RNHL); Hakkari, SW of Yüksekova, Varegös, Sat Dag (RNHL); Pamphylia, W of Alanya (ZMK).

**Distribution**. Bulgaria, Croatia, Czech Republic, France, Germany, Greece, Hungary, Iran, Iraq, Italy, Montenegro, Morocco, Poland, Romania, Russia (incl. Far East), Slovakia, Spain, Turkey, in addition to South Korea and Tajikistan (Eastern Palaearctic subregion).

#### 17. *Dryinus tigarae* Olmi

*Dryinus tigarae*Olmi 2008: 365.

**UNITED ARAB EMIRATES**: Abu Dhabi, Sweihan District, Al Ain (CNC) (Olmi 2008).

**Distribution**. United Arab Emirates.

#### 18. *Dryinus turcicus* Olmi

*Dryinus turcicus*Olmi 1991: 259; 1999: 199.

**TURKEY**: Hakkari District, Hakkari (RNHL) (Olmi 1991).

**Distribution**. Turkey.

#### 19. *Dryinus tussaci* Olmi

*Dryinus tussaci*Olmi 1991: 260.

**FRANCE**: Var, Vidauban (MNHN). **ITALY**: Sardegna, Sassari Prov., Berchidda, 40°47.99'N, 09°08.87'E (MOLC) (Olmi 2005b); Toscana, Grosseto Prov., Natural Park of Maremma, 42°38.17'N, 11°04.26'E (MOLC) (Olmi 2005b); Toscana, Pisa Prov., Monteverdi Marittimo, 43°09.59'N, 10°43.24'E (MOLC) (Olmi 2005b). **MOROCCO**: about 20 km N Agadir, Tarhazoute (MNHN) (Olmi 1991). **SPAIN**: Jaen, Alcaudete, Sierra del Ahillo (AMNH, MBC) (Olmi 1999).

**Distribution**. France, Italy, Morocco, Spain.

#### 20. *Dryinus yemenensis* Olmi and Van Harten

*Dryinus yemenensis*Olmi and van Harten 2006: 327.

**OMAN**: Dhofar, Salalah East, Dahariz, 17°01.02'N, 54°09.32'E (MOLC). **YEMEN**: Al Lahima (MOLC) (Olmi and van Harten 2006); 12 km NW of Manakhah (MOLC) (Olmi and van Harten 2006); Al-Kowd (MOLC) (Olmi and van Harten 2006).

**Distribution**. Oman, Yemen.

## Discussion

Dryininae of the Western Palaearctic subregion are insufficiently known from many points of view. The 20 listed species are known mainly on the basis of only one sex (Derafshan et al. 2016; Olmi 1999, 2008; Olmi and van Harten 2006; Olmi and Xu 2015). In fact, only females are known in 11 species (*D. berlandi*, *dayi*, *delvarei*, *gharaeii*, *gryps*, *ibericus*, *maroccanus*, *tigarae*, *turcicus*, *tussaci*, *yemenensis*). Both opposite sexes are known in six species (*D. balearicus*, *collaris*, *corsicus*, *niger*, *sanderi*, *tarraconensis*). In three species, the male was associated to the female tentatively, i.e. the association is doubtful (*D. albrechti*, *canariensis*, *tamaricicola*). This situation depends on the large morphological differences between female and male, so that the association of the opposite sexes is impossible, if it is not supported by rearings or DNA analysis. However, very few researchers rear dryinids or study their DNA.

The knowledge is insufficient also in the association of the species to their hosts. *Dryinus* species are parasitoids of Fulgoromorpha (Guglielmino et al. 2013). However, in the Western Palaearctic subregion, the hosts are known only in six species (*D. balearicus*, *collaris*, *corsicus*, *niger*, *sanderi*, *tarraconensis*). Also in this case, the situation depends on the scarcity of rearings.

From the biogeographical point of view, according to the categories presented by Vigna Taglianti et al. (1992, 1999, the chorotypes of the 20 species listed in the Western Palaearctic subregion are the following: *D. albrechti* (endemic, Macaronesian); *D. balearicus* (Western Mediterranean); *D. berlandi* (Western Mediterranean); *D. canariensis* (Mediterranean-Macaronesian); *D. collaris* (Turan-European); *D. corsicus* (Turan-European); *D. dayi* (endemic, Greece); *D. delvarei* (Eastern Mediterranean); *D. gharaeii* (endemic, Iran); *D. gryps* (Southern European); *D. ibericus* (endemic, Spain); *D. maroccanus* (Western Mediterranean); *D. niger* (European); *D. sanderi* (Turan-European); *D. tamaricicola* (endemic, Iran); *D. tarraconensis* (Asian-European); *D. tigarae* (endemic, United Arab Emirates); *D. turcicus* (endemic, Turkey); *D. tussaci* (Western Mediterranean); *D. yemenensis* (endemic, Yemen, Oman). Eight species of the above list are considered endemic provisionally, because dryinids are understudied, so their geographic distribution could be larger.

Olmi and Xu (2015) listed 10 species of *Dryinus* and one species of *Pseudodryinus* from the Eastern Palaearctic subregion. So the dryinid population of the two subregions has about the same numerical strength. However, the composition is different. Few species are present also in the Western Palaearctic subregion, i.e. *D. collaris*, *D. corsicus*, and *D. tarraconensis*.
